# Supplementary material for: Interaction of TLK1 and AKTIP as a Potential Regulator of AKT Activation in Castration-Resistant Prostate Cancer Progression
Source: Pathophysiology. 2021 Jul 20;28(3):339–54. doi: 10.3390/pathophysiology28030023 (PMC8830441; doi:10.3390/pathophysiology28030023)
Supplement: Supplementary file 1 [file pathophysiology-28-00023-s001.zip › pathophysiology-1247447-supplementary.pdf]

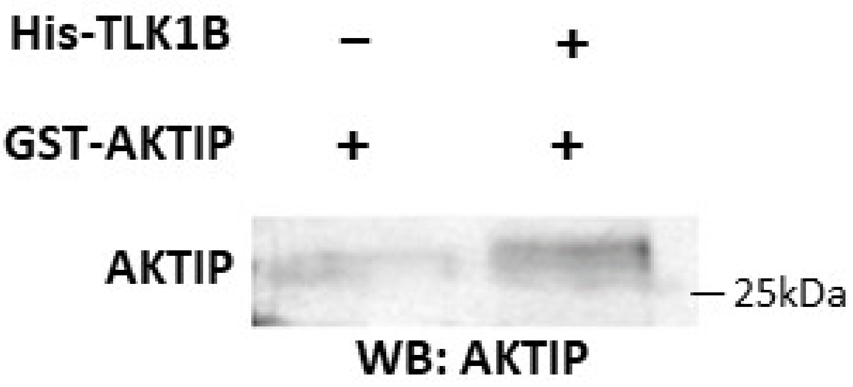

(**a**)

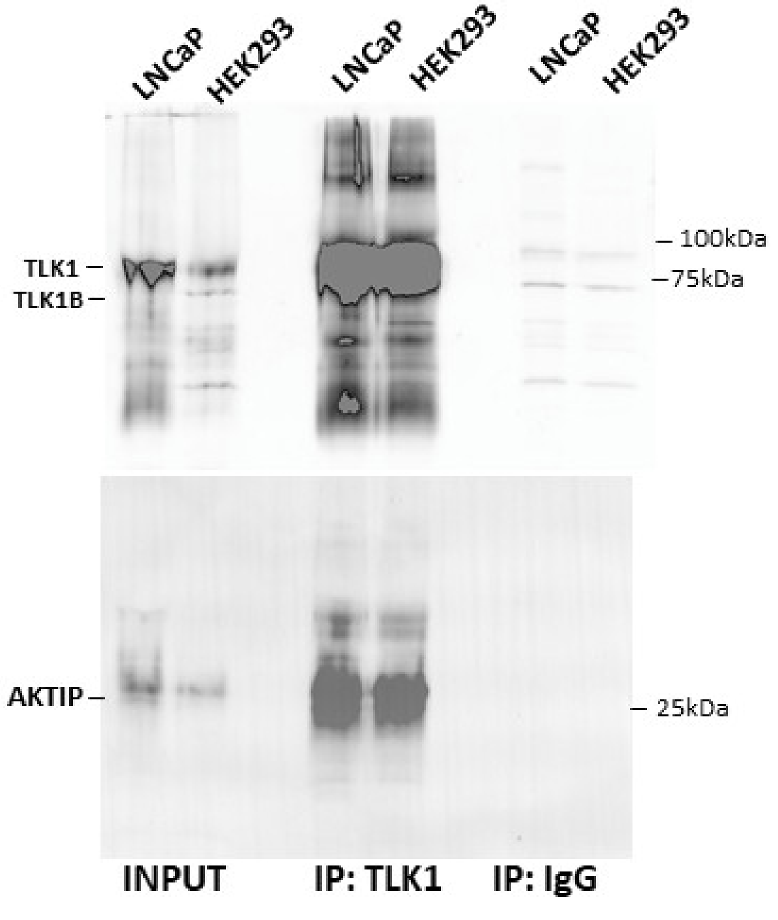

(**b**)

Figure S1. TLK1 and AKTIP interaction. (**a**) In vitro pull-down of recombinant His-TLK1B and GST-AKTIP isolated on Ni-NTA Sepharose and immunoblotting of AKTIP. (**b**) Co-IP of TLK1 from HEK 293 and LNCaP cell lysates using TLK1 specific antibody and immunoblotting for both TLK1/1B (upper panel) and AKTIP (lower panel). .
